# The Role of Automation for Early Diagnosis of Non-fermenter Superbugs in Critically Ill Septicemic Hospitalized Patients

**DOI:** 10.7759/cureus.41484

**Published:** 2023-07-06

**Authors:** Karvi Agarwal, Saurabh Agrawal, Naila Begum, Sonal Jindal

**Affiliations:** 1 Department of Microbiology, Lala Lajpat Rai Memorial Medical College (LLRMMC), Meerut, IND; 2 Department of Surgery, Lala Lajpat Rai Memorial Medical College (LLRMMC), Meerut, IND

**Keywords:** antimicrobial, non fermenters, bacteremia, non-fermenting gram-negative bacilli (nfgnb), bloodstream infections (bsi)

## Abstract

Introduction

Non-fermenting Gram-negative bacilli (NFGNB) are emerging superbugs of bloodstream infections (BSI), causing increased mortality in hospitalized patients. NFGNB are challenging to identify using conventional identification techniques. Hence, automation is beneficial for accurate and fast diagnosis; it also facilitates rapid treatment and recovery of patients. This study aims to isolate/identify NFGNB from BSI and determine its antimicrobial susceptibility pattern.

Material and methods

This study was conducted in the Department of Microbiology, LLRMMC, Meerut, for a period of six months (June to November 2022). The samples were processed using automated blood culture (BD BACTEC) and an identification/sensitivity testing system (BD Phoenix).

Results

Out of 1340 blood cultures, 347 (25.7%) were flagged positive for microbial growth. A total of 103 (7.6%) NFGNB were isolated, showing their strong association with BSI. The NFGNB isolates were *Pseudomonas species *23 (22.3%), *Acinetobacter baumannii* 19 (18.4%), *Salmonella *spp. 19 (18.4%), *Sphingomonas paucimobilis *17 (16.5%), *Aeromonas hydrophila *5 (4.8%), *Rhizobium radiobacter *sp. 4 (3.8%), *Stenotrophomonas maltophila *3 (2.9%), *Burkholderia cepacian *3 (2.9%), *Proteus mirabilis *2 (1.9%), *Achromobacter xylosoxidans* 2 (1.9%), *Elizabethkingia meningoseptica *2 (1.9%), *Ochromobacter anthropic *2 (1.9%), *Cuprivadus pauculus* 1 (0.9%), and *Ralstonia mannitolilytica* 1 (0.9%).

Conclusions

Automation helps in the prompt reporting of NFGNB and their antibiogram pattern by microbiology laboratories, facilitating the early and accurate management of patients with BSI.

## Introduction

Bacteremia is the presence of a viable bacterial agent in the bloodstream and is diagnosed in routine clinical practice using blood cultures. It leads to high healthcare costs and mortality, with a fatality rate as high as 30% [1]. Non-fermenting Gram-negative bacilli (NFGNB) are heterogenous, aerobic, ubiquitous, non-sporing saprophytic bacteria. They are termed "non-fermenters" because they do not utilize carbohydrates as an energy source through fermentation [2]. This heterogeneous group includes organisms like *Stenotrophomonas maltophilia*, *Acinetobacter* sp., *Pseudomonas* sp., *Alkaligenes* sp., *Burkholderia cepacian* complex, *Aeromonas hydrophila*, *Sphingomonas paucimobilis*, *Elizabethkingia meningoseptica*, *Ochromobacter anthropi*, *Ralstonia mannitolilytica*, and *Cuprivadus pauculus*. Most of them are nosocomial pathogens, causing opportunistic infections in immunocompromised patients [3].

NFGNBs are emerging superbugs of bloodstream infections (BSI), causing an increased rate of mortality in hospitalized patients. They invade and colonize the sterile site, which leads to the disruption of natural barriers. These organisms contribute to about 15% of all bacterial isolates in a clinical microbiology laboratory [4]. Data obtained from the Surveillance and Control of Pathogens of Epidemiological Importance (SCOPE) study revealed that approximately one-fourth of Gram-negative bacteremias were attributed to NFGNB [1]. They are difficult to identify using conventional techniques [1]. Hence, automation is beneficial for accurate and fast diagnosis to accelerate the treatment and recovery of patients, as it is a technology that completes the procedure without the assistance of any human [4].

Their multi-drug resistance (MDR) is a significant concern, which severely limits the treatment options. In low- or middle-income countries, a lack of standard antimicrobial guidelines, the emergence of antimicrobial resistance, the paucity of good diagnostic facilities, a poor hospital environment, and the quality of hand hygiene are significant determinants for the surge in NFGNB BSIs [5]. Therefore, early and precise identification with automation methods along with appropriate treatment is necessary to decrease the mortality and morbidity caused by these organisms in hospitalized patients [1].

The prevalence of NFGNB and their antibiogram has yet to be reported in this part of India, and this study was performed to bridge this gap in unawareness. It is the first report on the prevalence and antibiogram of NFGNB from Uttar Pradesh. This study aimed to isolate and recognize the NFGNB from blood specimens and determine their antimicrobial susceptibility pattern to improve the patient's outcome.

## Materials and methods

Setting

The study was conducted for six months (June to November 2022) in the Department of Microbiology in collaboration with the intensive care units (ICUs) of the tertiary care hospital of Lala Lajpat Rai Memorial (LLRM) Medical College, Meerut. One thousand three hundred forty samples received from various hospital departments were processed in the microbiology laboratory. Additionally, the Institutional Ethical Committee (IEC/LLRM/09/22) granted ethical approval, and all patients and patients' relatives gave their written consent to participate in the study. Patients of all age groups with signs and symptoms of BSI and septicemia, such as fever with or without chills, diaphoresis, tachypnea, tachycardia, leukocytosis, and leucopenia, and patients admitted to the ICU before the administration of any antimicrobial agent, were included in the study. However, non-septicemic patients and isolates other than NFGNB were excluded.

Sample collection and transport

About 5-10 mL of blood in adults and 1-5 mL in pediatric patients was collected in blood culture bottles (BD) on the patient's bedside under aseptic precautions after cleaning the site with 70% isopropyl alcohol. Bottles were sent to the microbiology laboratory immediately for culture. Samples were processed using automated blood culture (BD BACTEC) and an identification/sensitivity testing system (BD Phoenix M-50), and the results were interpreted per the Clinical and Laboratory Standard Institute (CLSI) 2022 guidelines.

Sample processing

Upon receipt in the lab, the blood culture bottle was directly placed into the BD FX-40 machine (BD), a fully automated blood culture system for detecting growth in blood. On all the bottles flagged positive, a Gram stain was carried out, followed by subculture on chocolate agar, 5% sheep blood agar, and MacConkey agar plates and aerobic incubation at 37 °C for 18-24 hours for bacterial isolation. Colony characteristics, Gram staining, rapid catalase and oxidase tests, and other biochemical reactions provisionally identified the non-fermenters. As per the manufacturer's instructions, an automated BD-Phoenix M-50 system performed the final identification and antimicrobial susceptibility testing.

## Results

Out of 1340 blood samples, 347 (25.7%) were flagged positive for microbial growth. NFGNB were isolated from 103 out of 347 positive samples, accounting for an isolation rate of 7.6%, showing a strong association with BSIs. Of these isolates, 68 (66%) were from male patients, and 35 (33.9%) were from female patients. The most common NFGNB isolates were *Pseudomonas* species 23 (22.3%), *Acinetobacter baumannii* 19 (18.4%), *Salmonella spp.* 19 (18.4%), *Sphingomonas paucimobilis* 17 (16.5%), *Aeromonas hydrophila* 5 (4.8%), *Rhizobium radiobacter* sp. 4 (3.8%), *Stenotrophomonas maltophila* 3 (2.9%), *Burkholderia cepacia* 3 (2.9%), *Proteus mirabilis* 2 (1.9%), *Achromobacter xylosoxidans* 2 (1.9%), *Elizabethkingia meningoseptica* 2 (1.9%), *Ochromobacter anthropi* 2 (1.9%), *Cuprivadus pauculus* 1 (0.9%), and *Ralstonia mannitolilytica* 1 (0.9%), as shown below (Table 1).

**Table 1:** Percentage distribution of various non-fermenter Gram negative bacterial isolates isolated from blood culture (n=103)

Bacterial Isolates	Number	Percentage
*Pseudomonas* spp.	23	22.33%
Acinetobacter baumannii	19	18.45%
*Salmonella* spp.	19	18.45%
Sphingomonas paucimobilis	17	16.50%
Aeromonas hydrophila	5	4.85%
*Rhizobium radiobacter* sp.	4	3.88%
Stenotrophomonas maltophila	3	2.92%
Burkholderia cepacia	3	2.92%
Proteus mirabilis	2	1.94%
Achromobacter xylosoxidans	2	1.94%
Elizabethkingia meningoseptica	2	1.94%
Ochromobacter anthropic	2	1.94%
Cuprivadus pauculus	1	0.97%
Ralstonia mannitolilytica	1	0.97%

Among the 103 isolates of NFGNB obtained from the ICUs, the maximum isolates were obtained from the neonatal ICU (30%) and the least from the pediatric ICU (20.3%), while the medical ICU and surgical ICU isolates were 29 (28.1%) and 22 (21.3%), respectively (Table 2).

**Table 2:** Percentage positivity of non-fermenter Gram negative bacteria isolated from various intensive care units of the hospital NICU: neonatal intensive care unit, MICU: medical intensive care unit, SICU: surgical intensive care unit, PICU: pediatric Intensive care unit

Ward	Number (n)	Percentage (%)
NICU	31	30.10
MICU	29	28.15
SICU	22	21.36
PICU	21	20.39

While obtaining the age-wise distribution of NFNGB, the maximum number of cases was observed in the pediatric age group of fewer than 28 days (44.6%), followed by the age groups 1 month to 10 years and 21-30 years (14.5%) (Table 3).

**Table 3:** Age-wise distribution of non-fermenter Gram negative bacteria

Age (years)	Number (n)	Percentage (%)
Less than 28 days	31	30.09
1 month to 10 years	15	14.56
11–20	11	10.68
21–30	15	14.56
31–40	9	8.74
41–50	6	5.83
51–60	6	5.83
>60	10	9.71

The antibiotic susceptibility test results of the NFGNB showed the percentage of resistance of various antibiotics tested against NFGNB isolates identified from blood culture. A high level of resistance was recorded among most of the isolates. The majority of the isolates showed good susceptibility to aminoglycosides and fluoroquinolones (Table 4).

**Table 4:** Antimicrobial resistance profile of the NFGNB (n=103) NFGNB: non-fermenter Gram negative bacteria, CFS: cefsulodine, PTZ: piperacillin/tazobactam, CPM: chlorpheniramine, CAZ: ceftazidime, AT: aztreonam, MRP: meropenem, IPM: imipenem, CL: chloramphenicol, AK: amikacin, GEN: gentamicin, CIP: ciprofloxacin

NFGNB	CFS	PTZ	CPM	CAZ	AT	MRP	IPM	CL	AK	GEN	CIP
Pseudomonas spp. (n=23) [sp. aeruginosa (17), sp. putida (4), sp. stutzeri (2]	6(26%)	6(26%)	10(38.4%)	11(47.8%)	10(38.4%)	8(34.7%)	8(34.7%)	5(21.7%)	7(30.4%)	7(30.4%)	8(34.7%)
Acinetobacter spp. (n=19)	19(100%)	19(100%)	19(100%)	19(100%)	19(100%)	19(100%)	19(100%)	Nil	8(42.1%)	11(57.8%)	19(100%)
*Sphingomonas paucimobilis* (n=17)	5(29.4%)	5(29.4%)	Nil	5(29.4%)	9(52.9%)	5(29.4%)	5(29.4%)	13(76.4%)	4(23.5%)	4(23.5%)	Nil
*Aeromonas hydrophila* (n=5)	Nil	3(60%)	2(40%)	2(40%)	1(20%)	1(20%)	1(20%)	Nil	1(20%)	1(20%)	1(20%)
*Rhizobium radiobacter* (n=4)	1(25%)	0	2(50%)	4(100%)	4(100%)	2(50%)	2(50%)	4(100%)	1(25%)	1(25%)	1(25%)
*Burkholderia cepacia* (n=3)	2(66.6%)	2(66.6%)	2(66.6%)	2(66.6%)	2(66.6%)	1(33.3%)	1(33.3%)	3(100%)	Nil	Nil	2(66.6%)
*Stenotrophomonas maltophilia* (n=3)	NA	NA	NA	3(100%)	All isolates were sensitive to minocycline, cotrimoxazole, and levofloxacin						
*Ochromobacter anthropi* (n=2)	Nil	Nil	Nil	2(100%)	2(100%)	Nil	Nil	2(100%)	Nil	Nil	Nil
*Elizabethkingia meningoseptica* (n=2)	Nil	Nil	2(100%)	2(100%)	2(100%)	Nil	Nil	2(100%)	Nil	Nil	Nil
*Achromobacter xylosoxidans* (n=2)	Nil	Nil	Nil	Nil	Nil	Nil	Nil	Nil	Nil	Nil	Nil
*Proteus mirabilis* (n=2)	Nil	Nil	2(100%)	2(100%)	2(100%)	2(100%)	2(100%)	2(100%)	Nil	Nil	Nil
*Ralstonia mannitolilytica* (n=1)	Nil	Nil	Nil	1(100%)	1(100%)	Nil	Nil	1(100%)	1(100%)	1(100%)	Nil
*Cuprivadus pauculus* (n=1)	Nil	Nil	Nil	Nil	Nil	Nil	1(100%)	Nil	1(100%)	1(100%)	Nil
Salmonella species (n=19)	NA	NA	19(100%)	All isolates were sensitive to azithromycin, chloramphenicol, and ofloxacin							19(100%)

## Discussion

NFGNBs are ubiquitous in the environment. They are now recognized as important healthcare-associated and opportunistic pathogens [6]. The correct and rapid identification of such bacteria in a clinical microbiology lab, along with antimicrobial susceptibility testing, is an essential step toward the effective treatment of septicemic patients. BSIs caused by NFGNB pose a challenge for clinicians and microbiologists because the laboratories have limited facilities for their identification and emerging antimicrobial resistance [4]. An increase in resistance to antimicrobials is common among NFGNB; few strains are resistant to commonly used antibiotics. MDR among these organisms makes the treatment complicated and expensive [6].

The present study was conducted to evaluate the prevalence of NFGNB in causing BSIs, especially in hospitalized critical care patients, and to know their antibiogram. Of the 1340 blood cultures, 347 (25.7%) tested positive for bacterial culture, and 103 (7.6%) grew NFGNB. Varied isolation rates were recorded in different studies. The results of our study were parallel with those conducted by Nazir et al., Sarwat et al., Juyal et al., Grewal et al., Manjunath et al., and Chang and Huang, who have reported a positivity rate of 9.3%, 11.2%, 11.6%, 16.9%, 28.3%, and 31.62%, respectively, in their studies [1,4,7,8-10]. Malini et al., in their study, reported a 4.5% isolation rate [11]. Another study by Rao and Shivananda reported a higher positivity rate of 66.88% [12]. These differences in isolation rates might be because of the different hospital infection control practices in various institutes. The prevalence rates in different studies compared with our study are depicted in Table 5.

**Table 5:** Isolation rates of NFGNBs in different studies conducted in various states of India NFGB: non-fermenter Gram-negative bacteria

Authors	Year	Place	Percent isolation
Malini et al. [11]	2009	Karnataka	4.5%
Juyal et al. [7]	2013	Uttarakhand	9.3%
Grewal et al. [8]	2017	Punjab	11.6%
Nazir et al. [1]	2019	Jammu and Kashmir	11.2%
Paul and Borah [13]	2020	Assam	16.8%
Sarwat et al. [4]	2021	Delhi	16.9%
Manjunath et al. [9]	2022	Karnataka	28.3%
Our Study	2022	Uttar Pradesh	7.6%

Pseudomonas spp. was the most common non-fermenter in all the studies, followed by Acinetobacter spp., which is consistent with our findings. The isolation rates of Pseudomonas species and Acinetobacter species in various studies are compared to our findings below (Table 6).

**Table 6:** Isolation rate of Pseudomonas spp. and Acinetobacter spp. in different studies in various states of India

Authors	Year	Place	Pseudomonas spp.	Acinetobacter spp.
Sidhu et al. [14]	2010	Amritsar	32.88	23.28
Samanta et al. [15]	2011	Chandigarh	26	66
Upgade et al. [16]	2012	Tamil Nadu	43	21
Nazir et al. [1]	2019	Jammu and Kashmir	9.1	73.3
Manjunath et al. [9]	2022	Karnataka	63.6	36.3
Our study	2022	Uttar Pradesh	22.3	18.4

In the current study, Salmonella spp. was isolated in 19 (18.4%) cases. Similarly, a study by Bhumbala et al. in Rajasthan reported an isolation rate of 9.1% [17]. In contrast, Patil and Mule reported a higher isolation rate of 76.5% [18].

Sphingomonas paucimobilis was isolated in 16.5% of cases in our study. The results are discordant with a study by Nazir et al., which reported a low prevalence rate of only 1.5% [1]. We isolated five cases of Aeromonas hydrophila in our study. However, the study done by Hirai et al. in Japan reported 24 cases of the same [19]. It indicates that the most common significant risk factors for Aeromonas bacteremia were solid tumors and complications associated with the liver, biliary tract, and pancreas, thereby indicating the immunocompromised state of the patient.

With respect to age, our study found a high prevalence of NFGNB among pediatric patients, particularly neonates (30%) and those under the age of 10 years (14.5%), followed by those in the age group 21-30 years (14.5%). Concordant results were reported in a study by Nazir et al. and Sarwat et al. [1,4]. This indicates that NFGNB plays a significant role in neonatal septicemia, causing the highest mortality in neonatal ICUs. Comorbidities that develop with age likely influence the invasiveness of NFGNB.

A broad range of nosocomial infections were caused by the NFGN [20,21]. Resistance patterns among nosocomial bacterial pathogens may vary demographically across various geographic regions [22]. Because of the high intrinsic resistance of different NFGNBs to different antimicrobial agents, proper identification and resistance testing are of paramount importance in a given setup to guide the appropriate selection of empiric therapy [1]. According to the antibiotic sensitivity pattern in the present study, it is evident that most of the isolates showed increased resistance against most of the first-line and second-line drugs, which confirms the MDR of NFGNB.

The antimicrobial susceptibility pattern of *Pseudomonas* spp. showed good susceptibility to colistin (78.3%), followed by piperacillin, tazobactam (74%), and amikacin (69.6%). It is in concordance with other reports where MDR in *Pseudomonas* spp. has been reported [23,24].

Other Gram-negative nonfermenters, such as *Rhizobium radiobacter*, *Stenotrophomonas maltophilia*, *Burkholderia cepacia*, *Achromobacter xylosoxidans*, *Elizabethkingia meningoseptica*, *Ochrmobacter antropi*, *Cuprivadus pauculus*, and *Ralstonia mannitolilytica*, were rarely isolated by other studies but showed vital significance in our study. During follow-up, it was found that there was a high mortality rate associated with these rare nonfermenters. One hundred percent mortality was observed in a patient with *Elizabethkingia meningoseptica* and in both patients with *Ochrmobacter antropi*. One patient infected with Ralstonia species also died after multisystem failure and septicemic shock, while three out of four patients with *Rhizobium radiobacter* died. Most of these patients were from the neonatal ICU, indicating a strong association between these infections and neonatal septicemia and mortality.

To determine the root cause of these deaths, we conducted environmental surveillance across various ICUs. Our investigation yielded remarkable results. Rhizobium, Pseudomonas, and Sphingomonas species were isolated from neonatal incubators, suction machines, and laryngoscopes. Despite the limitations of being conducted in a specific geographical area, this study emphasizes the importance of strictly complying with infection control practices in all ICUs. Essential tools of critical care, such as environmental surveillance, hand hygiene practices, PPE usage, and the implementation of the bundle approach, can help reduce and prevent ICU mortality.

Hence, this study serves as a wake-up call for all clinical microbiologists and clinicians to understand the importance of early and accurate diagnosis in patients with septicemia for faster and more effective treatment. Furthermore, automation in microbiology laboratories is a boon for the diagnosis and effective antimicrobial therapy of unusual pathogens like non-fermenters that are difficult to detect and often missed by traditional culture.

## Conclusions

Based on this study, we can conclude that non-fermenters, once considered contaminants, have arrived as potential pathological septicemic agents in critically ill patients. Automated systems are the need of the hour in microbiology laboratory setups that perform organism identification, and antimicrobial susceptibility testing is now a mainstay of clinical microbiology labs. Implementing the BD Phoenix instrument in our laboratory-based surveillance activities has allowed us to re-examine a subset of previously unidentified or misidentified NFGNB contributing to BSI. To improve patient outcomes, early identification of NFGNB is necessary due to varied sensitivity patterns. With the alarming increase in MDR acquired by non-fermenters, rendering many antimicrobial agents ineffective, clinicians must remain updated on the prevalence and antimicrobial susceptibility pattern of the circulating pathogens in order to select proper antimicrobials for empiric therapy. Therefore, it is crucial to have a proper antibiotic policy, carry out adequate screening of non-fermenters, regularly assess their antibiotic susceptibility profiles, and judiciously use antibiotics to effectively manage the infections caused by these organisms and limit the emergence of MDR. In our tertiary care hospital, antibiotic stewardship is still in its infancy; hence, we need to improve our infection control policies and protocols.
